# A Modeling Framework to Describe the Transmission of Bluetongue Virus within and between Farms in Great Britain

**DOI:** 10.1371/journal.pone.0007741

**Published:** 2009-11-05

**Authors:** Camille Szmaragd, Anthony J. Wilson, Simon Carpenter, James L. N. Wood, Philip S. Mellor, Simon Gubbins

**Affiliations:** 1 Institute for Animal Health, Pirbright Laboratory, Pirbright, Surrey, United Kingdom; 2 Cambridge Infectious Diseases Consortium, Department of Veterinary Medicine, University of Cambridge, Cambridge, United Kingdom; University of Leeds, United Kingdom

## Abstract

**Background:**

Recently much attention has been given to developing national-scale micro-simulation models for livestock diseases that can be used to predict spread and assess the impact of control measures. The focus of these models has been on directly transmitted infections with little attention given to vector-borne diseases such as bluetongue, a viral disease of ruminants transmitted by *Culicoides* biting midges. Yet BT has emerged over the past decade as one of the most important diseases of livestock.

**Methodology/Principal Findings:**

We developed a stochastic, spatially-explicit, farm-level model to describe the spread of bluetongue virus (BTV) within and between farms. Transmission between farms was modeled by a generic kernel, which includes both animal and vector movements. Once a farm acquired infection, the within-farm dynamics were simulated based on the number of cattle and sheep kept on the farm and on local temperatures. Parameter estimates were derived from the published literature and using data from the outbreak of bluetongue in northern Europe in 2006. The model was validated using data on the spread of BTV in Great Britain during 2007. The sensitivity of model predictions to the shape of the transmission kernel was assessed.

**Conclusions/Significance:**

The model is able to replicate the dynamics of BTV in Great Britain. Although uncertainty remains over the precise shape of the transmission kernel and certain aspects of the vector, the modeling approach we develop constitutes an ideal framework in which to incorporate these aspects as more and better data become available. Moreover, the model provides a tool with which to examine scenarios for the spread and control of BTV in Great Britain.

## Introduction

The advent of increased computing power over the past decade has facilitated the development of national-scale micro-simulation models for the transmission of livestock diseases that can be used to examine the potential spread of epidemics and assess the impact of control measures. This type of approach came to particular prominence during the 2001 foot-and-mouth disease (FMD) epidemic in Great Britain (GB) [1]–[5]. Since then, large-scale micro-simulation models have been developed for a range of livestock diseases, species and countries: for example, avian influenza in poultry in GB [6]–[8], scrapie in sheep in GB [9]–[11] and classical swine fever in pigs in the Netherlands [12].

To date, however, little attention has been given to the development of similar models for vector-borne diseases of livestock, such as bluetongue. Bluetongue (BT) is a non-contagious, infectious, insect-borne disease of ruminants caused by bluetongue virus (BTV) and is transmitted between hosts by the bites of *Culicoides* midges. Over the past decade BT has become one of the most important diseases of livestock following a series of incursions into Europe, largely under the influence of climate change [13]–[15]. In particular, the first cases of BTV serotype 8 (BTV-8) in northern Europe were reported near Maastricht in the Netherlands in August 2006, with subsequent cases reported in Belgium, Germany, France and Luxembourg [16]. In May 2007, BTV-8 re-emerged and caused major outbreaks across the previously-affected countries, and spread into new areas, including to the south-east of GB in the autumn of that year.

The aim of this paper is to present a modelling framework to describe the transmission of BTV within and between farms in GB, which can be used to predict patterns of spread following an incursion of BTV in British livestock and to assess the impact of different control measures, particularly vaccination. The model is stochastic and spatially-explicit with two components: the first describes the spread within a farm, and is parameterised using published data including explicit temperature-dependence where this has been quantified [17]; the second describes the spread between farms using a transmission kernel, estimated using data from the 2006 BTV epidemic in northern Europe. Once parameterised, the model was validated using data on the spread of BTV in GB during 2007. Finally, sensitivity analyses were carried out, particularly with respect to the shape of the transmission kernel.

## Methods

### Transmission of BTV within farms

**Figure 1:**
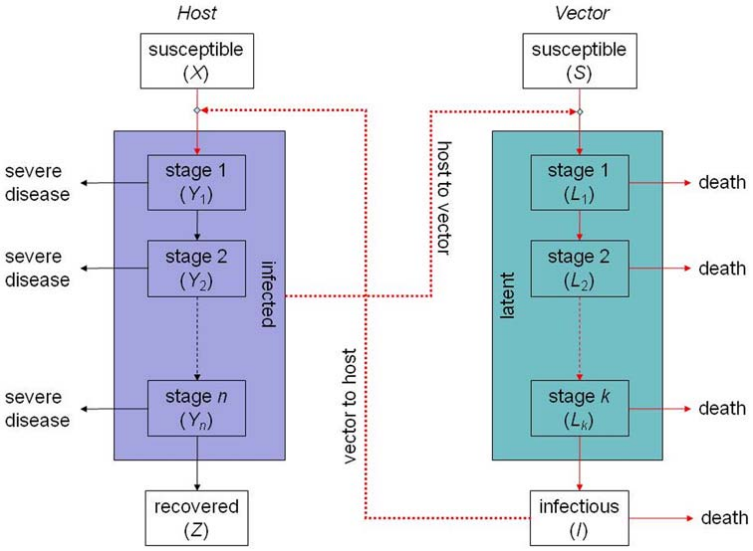
Schematic diagram of the model for the transmission dynamics of BTV within a farm. The populations of infected hosts and vectors are subdivided into a number of stages to allow for more general distributions for the duration of viraemia and the extrinsic incubation period, respectively. A solid line indicates a flow from one compartment to another; a dotted line indicates that a compartment has an influence on a rate of transfer. Lines shown in red indicate a temperature-dependent rate.

The transmission dynamics of bluetongue virus (BTV) within a holding are described using a stochastic compartmental model that includes two ruminant host species (cattle and sheep) and the *Culicoides* vector (Figure 1; Table 1), and was developed from an earlier deterministic formulation of the model [17]. The cattle and sheep populations are assumed to be constant (*H_i_*), except for disease-associated mortality, and are subdivided into the number of susceptible (i.e. uninfected), infected and recovered animals, denoted by *X* ^(*i*)^, *Y* ^(*i*)^ and *Z* ^(*i*)^, respectively, where the superscript *i* indicates cattle (*C*) or sheep (*S*). To allow for a more general distribution for the duration of viraemia, the infected host population, *Y* ^(*i*)^, is subdivided into a number of stages, with newly infected hosts entering the first stage and then passing through each successive stage. If the mean time spent in each stage is 1/*nr*, the total length of time spent in the *n* stages follows a Gamma distribution, with mean 1/*r* and variance 1/*nr* ^2^. The vector population (*N*) is subdivided into the number of adult female midges that are susceptible (i.e. uninfected), latent (i.e. infected, but not infectious) and infectious, denoted by *S*, *L* and *I*, respectively. To allow for a more general distribution for the extrinsic incubation (i.e. latent) period (EIP), the latent class is subdivided into a number of stages in a similar approach to that described above for the duration of host viraemia. Once infectious, midges remain so for life. Adult males and immature (larval and pupal) stages are not considered as they do not blood-feed and, hence, do not transmit BTV.

**Table 1:** Transitions, probabilities, and population sizes in the model for the transmission of BTV within farms†.

description	transition	probability	population size
*host population*			
infection	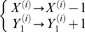	λ*_i_*δ*t* (see equation (1))	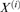
completion of infection stage *j* (*j* = 1,…,*n_i_*-1)	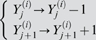	*n_i_r_i_*δ*t*	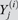
disease-associated mortality (*j* = 1,…,*n_i_*)	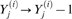	*d_i_*δ*t*	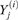
recovery	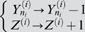	*n_i_r_i_*δ*t*	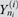
*vector population*			
Recruitment	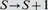	see Table 2	–
Infection	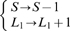	λ*_V_*δ*t* (see equation (3))	*S*
completion of EIP stage *j* (*j* = 1,…,*k*-1)	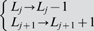	*K*νδ*t*	*L_j_*
vector mortality (*j* = 1,…,*k*)	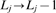	μδ*t*	*L_j_*
completion of EIP	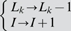	*K*νδ*t*	*L_k_*
vector mortality	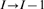	μδ*t*	*I*

† Parameters are defined in equations (1)–(3) and Table 2.

The force of infection for each host species, λ*_i_*, is given by,
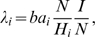
(1)which is the product of the probability of transmission from an infected midge to a host (*b*), the biting rate on the species (*a_i_*), the ratio of vectors to hosts (*m_i_* = *N*/*H_i_*) and the proportion of bites which are from infectious midges (*I*/*N*). The biting rate on species *i* can be decomposed such that *a_i_* = *a*φ*_i_*, where *a* is the reciprocal of the time interval between blood meals and φ*_i_* is the proportion of bites on the species. The proportion of bites on cattle (φ*_C_*) is given by,



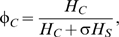
(2)while that on sheep is φ*_S_* = 1-φ*_C_*. The parameter σ is a measure of vector preference for cattle compared to sheep: if σ<1, vectors feed preferentially on cattle, while if σ>1, they feed preferentially on sheep. The force of infection for vectors, λ*_V_*, is given by,



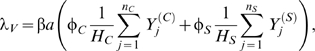
(3)which is the product of the probability of transmission from an infected host to a midge (β), the biting rate on hosts of each species, and the proportion of hosts that are infected.

Bluetongue was assumed to be detected on a farm if an animal died due to disease or if overt clinical signs were observed in at least one animal. This occurred in species *i* with daily probability given by,



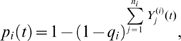
(4)where *q_i_* is the probability of detecting overt clinical signs in an infected animal of species *i*, and 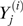
is the number of animals of species *i* in infection stage *j* at time *t*.

Population sizes in the model 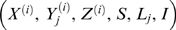
all take integer values and transitions (for example, infection, completion of an infection stage or death; Figure 1) are stochastic processes as summarised in Table 1. The number of transitions of each type which occurs during a small time interval δ*t* was drawn from a binomial distribution B(*n*,*q*) where *n* is the population size and *q* is the transition probability (the appropriate *per capita* rate multiplied by δ*t*; Table 1). However, binomial random variables are computationally expensive to simulate and an approximating distribution was used wherever possible. When one of the following conditions was satisfied, (i) *nq*(1-*q*)>25, (ii) *nq*(1-*q*)>5 and 0.1<*q*<0.9, or (iii) *nq*>10 and *n*(1-*q*)>10, an approximating normal variate with mean *nq* and variance *nq*(1-*q*) was used, while if *q*<0.1 and *nq*<10, an approximating Poisson variate with mean *nq* was used [18]. Those probabilities which include temperature-dependent parameters (see Table 2) were computed using hourly temperature data for the farm.

**Table 2:** Parameters in the model for the within-farm transmission of BTV.

description	symbol	estimate or range	comments	references
probability of transmission from vector to host	*b*	0.8–1.0	–	[39]
probability of transmission from host to vector	β	0.001–0.15	–	[40], [41], [42], [43]
biting rate on species *i*	*a_i_*	–	can be decomposed so that *a_i_* = *a*φ*_i_*	–
reciprocal of the time interval between blood meals	*a*	0–0.5	depends on temperature θ: *a*(θ) = max(0,0.0002θ(θ−3.7)(41.9−θ)^1/2.7^) [44]	[44], [45], [36]
vector preference for cattle compared to sheep	σ	0–1	vectors feed preferentially on cattle based on data for *C. imicola*	[46], [47]
number of cattle on holding	*H_C_*	–	obtained from the 2006 June agricultural survey for each holding	–
number of sheep on holding	*H_S_*	–	obtained from the 2006 June agricultural survey for each holding	–
duration of viraemia (cattle) - mean	1/*r_C_*	20.6	duration of viraemia based on natural infection and virus isolation in embryonated chicken eggs;	[48]
duration of viraemia (cattle) - no. stages	*n_C_*	5	parameters estimated by fitting a gamma distribution to data presented in paper cited in right-hand column	[48]
disease-induced mortality rate (cattle)	*d_C_*	0–0.0001	cattle seldom succumb to severe disease; upper limit derived from the BT outbreak in northern Europe in 2006 and 2007 where mortalities of up to 0.2% were observed	[16], [48], [49], [50]
probability of overt clinical signs (cattle)	*q_C_*	0.0078–0.067	–	[51], [50]
duration of viraemia (sheep) - mean	1/*r_S_*	16.4	duration of viraemia based on experimental infection and virus isolation in embryonated chicken eggs;	[52], [53]
duration of viraemia (sheep) - no. stages	*n_S_*	14	parameters estimated by fitting a gamma distribution to data presented in papers cited in right-hand column	[52], [53]
disease-induced mortality rate (sheep)	*d_S_*	0.001–0.01	derived from observed mortality in sheep ranging from 3.9% to 14.4%	[16], [54], [50]
probability of overt clinical signs (sheep)	*q_S_*	0.027–0.080	–	[51], [50]
vector recruitment rate	ρ	–	for simplicity assumed to be equal to the vector mortality rate	–
vector population size	*N*	see comments	based on a maximum host biting rate (*a_i_N*/*H_i_*) of 2500 bites per host per day and, hence, a vector to host ratio (*m_i_* = *N*/*H_i_*) of 0–5000; the ratio of vectors to cattle (or sheep if there were no cattle on the farm) was sampled from this range and the vector population size was computed such that *N* = *m_i_H_i_*	[40], [36]
extrinsic incubation period (EIP) - mean	1/ν	–	depends on temperature θ:ν(θ) = max(0,0.0003θ(θ−10.4)) [44]	[44], [55], [56]
extrinsic incubation period (EIP) - no. stages	*k*	1–100	–	[44, 55, 56
vector mortality rate	μ	–	depends on temperature: μ(θ) = 0.009exp(0.16θ) [55]; this is comparable with estimates of the mortality rate for *C*. *obsoletus* and *C*. *pulicaris* group midges derived using unpublished light-trap data from Pirbright, UK	[57], [55], [56]

### Transmission of BTV between farms

To describe the spread of BTV between farms, a stochastic, spatially-explicit model with a daily time-step was used. The unit of population was the farm, with farms divided into four classes: susceptible (no hosts or vectors infected with BTV); exposed (has acquired infection); infectious (has acquired infection and the first newly infected vectors on the farm have completed their extrinsic incubation period); and recovered (no longer any hosts or vectors infected with BTV).

Transmission between farms was described by a generic mechanism, which implicitly includes transmission via the movement of both vectors and host animals. The probability that an unaffected farm *j* acquires infection on day *t* is given by,



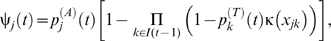
(5)where *I*(*t*) is a list of infectious farms on day *t*, 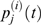
is the probability of acquisition (*A*) or transmission (*T*) for farm *j* on day *t*, respectively, and κ(*x_jk_*) is the transmission kernel, with *x_jk_* the (Euclidean) distance between farms *j* and *k*. The probabilities of acquisition and transmission were parameterised as,



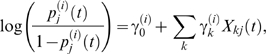
(6)where *X_kj_*(*t*) is the value of risk factor *k* for farm *j* on day *t* (for example, presence of cattle or sheep, temperature) and 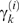 is the parameter associated with factor *k*.

Once a farm acquired infection, the within-farm dynamics were simulated as described above, based on the number of cattle and sheep kept on the farm and local temperatures. This was used to determine the time to infectiousness (defined as the time until the appearance of the first newly infectious vectors on the farm), the time to appearance of clinical signs (assumed to occur if an animal died due to disease or if overt clinical signs were observed in at least one animal), and the duration of the outbreak (defined as the time after which there are no more infected hosts or vectors on the farm).

### Parameter estimation: within-farm dynamics

Parameter estimates for the within-farm model (Table 1; Figure 1) have been previously derived from the published literature as part of uncertainty and sensitivity analyses of the basic reproduction number for BTV [17] and these estimates were used in the transmission model (Table 2). Temperature-dependent functions were used for the reciprocal of the time interval between blood meals (*a*), the vector mortality rate (μ) and the extrinsic incubation period (1/ν). Point estimates for parameters relating to the duration of host viraemia (*r_i_* and *n_i_*) were obtained by fitting Gamma distributions to published data. Finally, plausible ranges were determined for the remaining parameters to reflect uncertainty in their values; these parameters were set for each within-farm outbreak by sampling uniformly from these ranges.

### Parameter estimation: between-farm dynamics

Parameters in the model for the transmission of BTV between farms, including the form for the transmission kernel, were estimated from data on BTV-8 outbreak in northern Europe during 2006 [16], [19]. The data were obtained from the European Commission Animal Disease Notification System (ADNS) database, and included farm locations, farm type (cattle only, sheep only or mixed cattle and sheep) and the date of first clinical suspicion. Parameter estimation was performed using a dataset including all farms for which the date of first clinical suspicion was reported to be between 5 July 2006 and 1 November 2006 (inclusive). For simplicity, farms were assumed to become infected on the date of first clinical suspicion and infectious on the date after the first clinical suspicion. Once infectious, farms were assumed to remain so until at least the last day in the data-set.

Because data were available only for affected farms in the northern European outbreak, parameter estimation was done by maximising the log-likelihood conditional on a farm becoming infected at some point during the outbreak [20], which is given by,



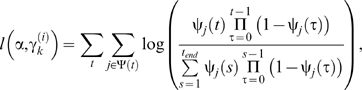
(7)where ψ*_j_*(*t*) is the probability that farm *j* acquires infection on day *t* (defined by equation (5)), Ψ(*t*) is a list of farms which acquire infection on day *t*, and *t_end_* is the last day in the data-set.

A number of transmission models, (5), were fitted to the data using the conditional log-likelihood, (7). Three forms for the transmission kernel were considered, namely, exponential (*E*), Gaussian (*G*) and fat-tailed (*F*) forms, which are given by,



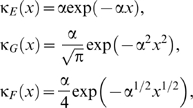
(8)respectively, where α is the kernel parameter. Probabilities of acquisition and transmission (see equation (6)) including or excluding the presence of cattle and the presence of sheep (Table 3) and daily mean temperature (as linear and quadratic terms) were considered. The models were compared using the Akaike information criterion (AIC), with a difference in AIC of two assumed to indicate a significant difference between models.

**Table 3:** Summary of demographic factors included in models for the probability of transmission of BTV between farms.

model	probability of acquisition		probability of transmission	
	*presence of cattle*	*presence of sheep*	*presence of cattle*	*presence of sheep*
1	**YES**	**YES**	**YES**	**YES**
2	**YES**	**YES**	**YES**	No
3	**YES**	**YES**	no	**YES**
4	**YES**	no	**YES**	**YES**
5	no	**YES**	**YES**	**YES**
6	**YES**	no	**YES**	no
7	no	**YES**	no	**YES**
8	**YES**	no	no	**YES**
9	no	**YES**	**YES**	no
10	**YES**	no	no	no
11	no	**YES**	no	no
12	no	no	**YES**	no
13	no	no	no	**YES**
14	no	no	no	no

### Simulating the dynamics of BTV-8 in GB

The location and the number of sheep and cattle on each holding were obtained from June agricultural survey data for 2006. Hourly temperature records for 2007 were extracted from the BADC/MIDAS database [21] for 19 meteorological stations throughout GB (see Figure S1 and Figure S2).

The model was initialised with a single infected farm (Baylham Farm, near Ipswich) on 4 August 2007. This has been identified as the most likely day of introduction of infected midges from the near continent [22]. Six additional farms became infected later (two in Cambridgeshire, three in Kent and one in Sussex), with no demonstrable epidemiological link to the main East Anglian focus. It has been suggested that these additional cases may have been a result of new introduction events. They were therefore included as additional seeds in the simulations, based on their location and date of reporting.

Restrictions on animal movements are imposed in response to an outbreak of bluetongue, with movements not allowed out of a designated protection zone (PZ) except under certain circumstances. The impact of these movement restrictions was assessed by simulating the model with either no restrictions on transmission between farms or by allowing transmission only between farms in the 2007 PZ declared by Defra [23]. This is the simplest means of incorporating the effect of movement restrictions in the model and, moreover, assumes that most long-distance transmission is the result of animal movements rather than vector dispersal, which is likely to be the case.

For each scenario the model was run so that 50 outbreaks (defined as any increase in the number of affected holdings beyond those seeded in the simulations; see above) were generated. Replicates were simulated until the required number of outbreaks (50) had been generated, so that the number of replicates was not specified in advance, but follows a negative binomial distribution. The number of outbreaks was chosen to provide robust results without being prohibitively expensive in terms of computation time.

## Results

### Parameter estimation: between-farm dynamics

**Figure 2:**
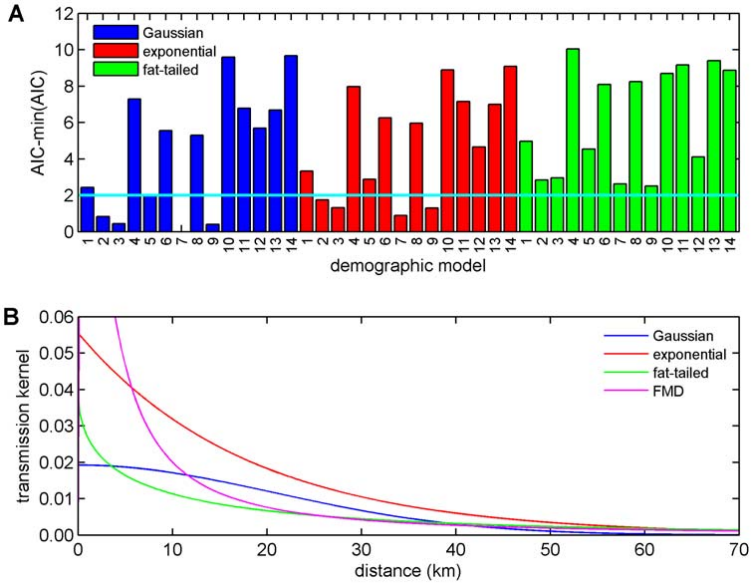
Models for the probability of transmission between farms. (A) Comparison of model fit for different transmission kernels and demographic models (defined in Table 3) based on the Akaike information criterion (AIC). The cyan line indicates a difference of two in AIC between a model and that with the lowest AIC (i.e. Gaussian kernel and demographic model presented in Table 4), taken to represent a significant difference in model fit. (B) Transmission kernels, (8), using the maximum-likelihood estimates obtained by fitting the models to outbreak data from northern Europe in 2006 (Table 4). The FMD kernel is that estimated by Chis Ster and Ferguson [24] from the outbreak of foot-and-mouth disease (FMD) in the UK during 2001.

Three kernels (see equation (8)) and 14 demographic models (Table 3) (i.e. a total of 42 models) were fitted to data on the BTV-8 outbreak in northern Europe in 2006. Comparison of the transmission kernels indicates that a Gaussian kernel yields the best fit for a given demographic model, followed by an exponential kernel, with a fat-tailed kernel yielding the poorest fit (Figure 2A). Moreover, the fat-tailed kernel always yielded a significantly poorer fit than either a Gaussian or exponential kernel (Figure 2A). The best-fit kernels are shown in Figure 2B (see also Table 4) together with the kernel estimated from the 2001 outbreak of foot-and-mouth disease (FMD) in the GB [24], [25]. The key difference between the kernels is the thickness of the tails (i.e. the probability of transmission at longer distances), which are much higher for the fat-tailed and FMD kernels than for the Gaussian or exponential ones (Figure 2B).

**Table 4:** Maximum-likelihood estimates for the probability of transmission between farms when fitted to data on the spread of BTV in northern Europe during 2006.

parameter	kernel		
	Gaussian	exponential	fat-tailed
transmission kernel	0.034	0.056	0.161
*probability of acquiring infection*			
intercept	0.562	−0.516	1.268
presence of sheep on farm	−1.330	−0.958	−1.755
*probability of transmitting infection*			
intercept	−2.149	−2.031	−1.499
presence of sheep on farm	30.095	30.24	31.126
Akaike information criterion (AIC)	743.11	744.00	745.73

The ranking of the demographic models by the AIC is similar for each of the kernels, with a model including the presence of sheep as a factor in both the probability of acquisition and transmission (model 7; Table 3) providing the best fit to the data (Figure 2A). Furthermore, the parameter estimates in the demographic models for the different kernels are comparable, especially for the effect of the presence of sheep on the probability of transmission (Table 4).

In addition to the effects of farm demography on the probability of transmission between farms, we also explored the impact of temperature on both the probabilities of acquisition and transmission, (6), by including it as linear and quadratic terms. However, no significant improvements in model fit were identified for any of the models including temperature. Moreover, there were also problems with identifying robust estimates for any of the temperature-related parameters. Accordingly, temperature was not included as a factor in the probability of transmission between farms. Rather the influence of temperature was incorporated via its effects on the dynamics of BTV within a holding.

### Dynamics of BTV-8 in Great Britain during 2007

Simulating the dynamics of BTV-8 in GB following an incursion into East Anglia suggested that only a small proportion of incursions resulted in outbreaks (2.8%; 95% confidence interval (CI): 2.6–3.1%). This proportion did not differ significantly amongst kernels with or without movement restrictions (χ^2^ = 6.15, df = 7, *P* = 0.52).

**Figure 3:**
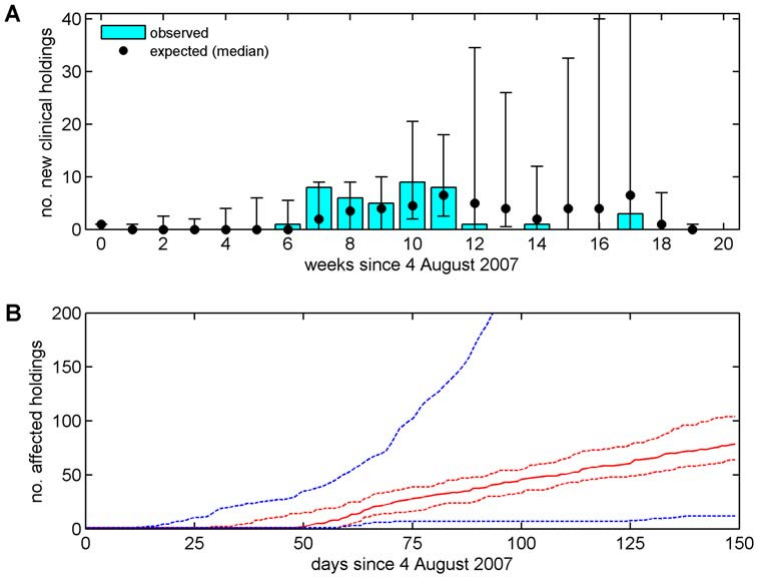
Temporal dynamics of BTV-8 in GB during 2007. (A) Observed and expected number of farms reporting clinical disease each week. The figure shows the observed number of newly-identified holdings with confirmed clinical cases (bars) and the median (symbols) and 10th and 90th percentiles (error bars) for the simulated outbreaks. (B) Expected cumulative number of affected holdings over time. The figure shows the median (solid red line), 25th and 75th percentiles (red dashed lines), and 10th and 90th percentiles (dashed blue lines). Each figure shows the results for the simulated epidemics assuming a Gaussian transmission kernel and demographic model presented in Table 4 (i.e. the best-fit model to the northern European data), based on the results of 50 simulated outbreaks which took off (see Methods).

Comparing the observed and expected time-course for farms reporting clinical disease in 2007 shows that the model captures the dynamics of the outbreak, including the delay of five to six weeks between the initial introduction of BTV and the first reported cases, followed by the rise and fall in the number of farms reporting cases over subsequent weeks (Figure 3A). Although the median number of farms reporting cases captured well the observed dynamics, there were a number of outbreaks for which the predicted number of clinically-affected holdings in 2007 was substantially higher than observed. This is primarily a consequence of the uncertainty in the model parameters, notably relating to vector abundance (Table 2), as well as the inherent stochasticity of infection dynamics. The median for the cumulative number of affected holdings (Figure 3B) underestimated the observed number (125) of affected holdings in 2007 (i.e. those detected by any surveillance method: reported cases, serosurveillance or pre-movement testing; [26]). However, the observed number of affected holdings is consistent with model predictions (Figure 3B).

**Figure 4:**
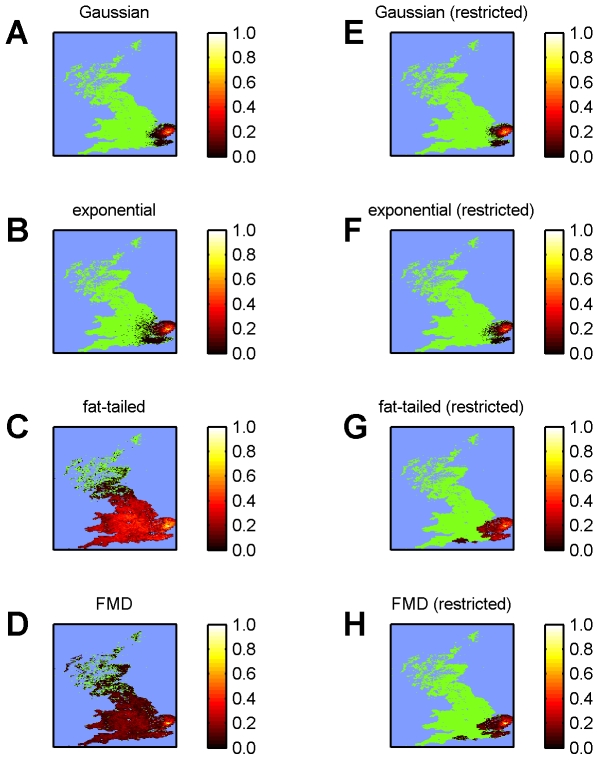
Spatial dynamics of BTV-8 in GB during 2007. Predicted spatial distribution of affected farms as of 31 December 2007 assuming: (A,B) a Gaussian kernel; (C,D) an exponential kernel; (E,F) a fat-tailed kernel; or (G,H) the FMD kernel. Transmission between farms is either (A,C,E,G) unrestricted or (B,D,F,H) restricted to the 2007 PZ. Each map shows the cumulative risk (see colour bars) expressed as the proportion of simulated outbreaks (out of 50 which took off; see Methods) for which at least one farm was affected by BTV within each 5 km grid square.

The predicted spatial distribution of clinically-affected holdings (Figure 4A and 4B) broadly corresponds to the observed distribution of reported cases, with the areas of highest risk in East Anglia matching the location of known affected holdings. Furthermore, the area at risk also broadly corresponds to the PZ put in place by Defra [23].

### Sensitivity analysis

**Figure 5:**
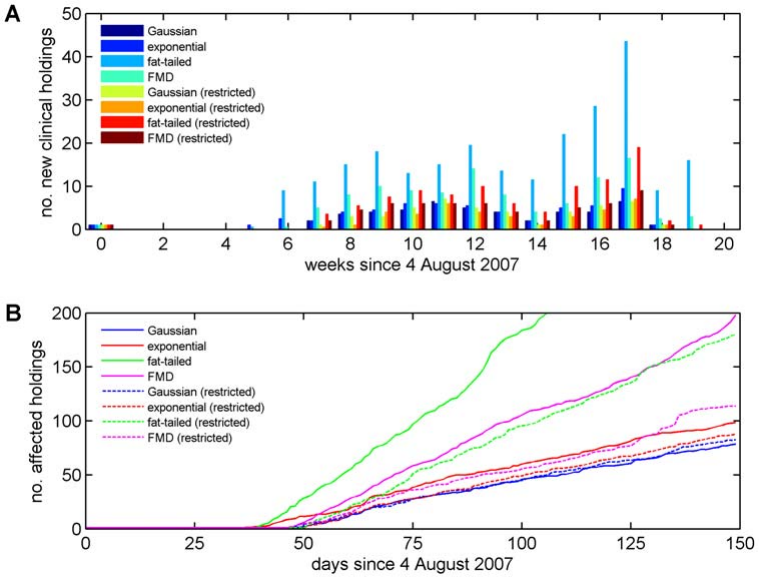
Sensitivity of temporal dynamics of BTV-8 to different transmission kernels. Comparison of (A) the median number of newly-identified holdings with confirmed clinical cases and (B) the cumulative number of affected holdings over time for different transmission kernels, where transmission between farms is either unrestricted or restricted to the 2007 protection zone. The figures are based on the results of 50 simulated outbreaks which took off (see Methods).

The sensitivity of the model predictions to the shape of the kernel was assessed by simulating outbreaks of BTV-8 in GB using four kernels: Gaussian, exponential, fat-tailed and FMD (Figure 2B; see also Table 4). Comparison of the model predictions for the different kernels shows that the Gaussian and exponential kernels result in similar time-courses and spatial dynamics (Figure 4A–4D and Figure 5). By contrast both the fat-tailed and FMD kernels predict consistently higher numbers of clinical and affected holdings (Figure 5) and a considerably greater extent of spatial spread, especially so for the fat-tailed kernel (Figure 4E and 4G).

Incorporating the effect of movement restrictions on transmission between farms had little effect on the model predictions using a Gaussian kernel (Figure 4A and 4B and Figure 5) and only a small effect on those using an exponential kernel (Figure 4C and 4D and Figure 5). By contrast, there was a marked effect on the predictions using a fat-tailed or the FMD kernel. In the case of a fat-tailed kernel, the model still predicted higher numbers of clinical and affected holdings than were observed, though to a lesser extent than without restrictions (Figure 4E and 4G and Figure 5), while in the case of the FMD kernel, the model predictions more closely matched those with either the Gaussian or exponential kernels (Figure 4G and 4H and Figure 5).

## Discussion

This paper presents a modelling framework for the transmission of BTV within and between farms in GB, which can be used to predict patterns of spread and, in particular, assess the impact of different control strategies, especially those involving vaccination. The model was constructed using available data from published literature and from the outbreak of BTV-8 in northern Europe during 2006 and was then validated using data on the spread of BTV-8 in GB during 2007.

For simplicity and because of the limited outbreak data available with which to parameterise the model, the spread of BTV between farms was described using a generic transmission kernel, (5), which implicitly includes all modes of transmission between farms and, in particular, the movement of animals and dispersal of vectors. This is a common approach in epidemiological modelling and has been widely used for other animal diseases, notably FMD [1], [24] and avian influenza [6]. Importantly, the choice of kernel and, in particular, the thickness of the tails (i.e. the probability of long-range transmission events) will have an impact on the predictions for spread [27], [28].

Previous analyses of directly-transmitted viruses and, in particular, those of FMD virus in GB have suggested that a power-law kernel (i.e. a fat-tailed kernel) is most appropriate to describe the spread of these viruses [24], [25]. In our analyses of BTV, a vector-borne infection, we explored a number of possible shapes for the kernel (exponential, Gaussian and fat-tailed), of which a Gaussian form yielded the best fit of the model to data on the spread of BTV-8 in northern Europe during 2006 (Figure 2A ; Table 4). Moreover, a Gaussian kernel captured the observed spread of BTV-8 in GB during 2007 (Figure 3A and Figure 4A), as did an exponential kernel (Figure 4B and Figure 5B), while a fat-tailed kernel or that derived for FMD did not (Figure 4E and 4G and Figure 5). If, however, the effect of movement restrictions was incorporated in the model, the predictions using the FMD kernel did match the observed dynamics of BTV in 2007 more closely (Figure 4H and Figure 5).

This raises two important issues. First, it is likely to be difficult to identify the shape of the kernel when transmission is impacted by control measures. At first glance, there are clear differences in the ability of the models with different kernels to capture the observed dynamics of BTV. However, once the effect of movement restrictions are included, the differences become less clear. Consequently, it is essential to consider the sensitivity of model predictions for spread or the impact of control measures to the shape of the transmission kernel. Second, any assessment of the efficacy of movement restrictions at preventing the spread of BTV will depend critically on the shape of the transmission kernel (see also [29]). In particular, our results for a Gaussian kernel would suggest they have little impact on spread and are not necessary, while those using the FMD kernel would suggest movement restrictions are effective at reducing spread.

Two other studies have considered the spread of BTV-8 in northern Europe using either random walk [30] or wind density [31] models. The conclusion when describing spread as a random walk was that the data were adequately described by a Gaussian model [30], as was the case with our analysis (Figure 2A ; Table 4). The wind density model indicated that transmission over short distances (<5 km) was symmetric, but identified asymmetries for spread over medium (5–31 km) or long distances (>31 km) [31]. Although the wind density approach is potentially useful for retrospective analyses, it focuses on a single mechanism of spread (i.e. vector dispersal by wind). Furthermore, any asymmetries will be a consequence of a range of location-specific factors (for example, topography in the case of wind). Incorporating this level of detail would increase substantially the model's complexity without greatly adding to its utility for exploring the spread and control of BTV in GB or, indeed, elsewhere.

There is likely to have been under-ascertainment of affected holdings in the northern European data for 2006, partly through under-reporting, but also because clinical signs of disease could be missed or, indeed, not present. The impact of under-ascertainment was assessed by simulating outbreak data using the model, (5), selecting a percentage (10–100%) of affected farms at random and then using this sampled, simulated data-set to estimate parameters as described in the Methods. Reasonable estimates (i.e. the true values lies within in the 95% confidence limits) for the transmission kernel parameter were obtained provided at least 50% of affected holdings were identified. However, the overall effect of under-ascertainment is to lead to an under-estimation of the spatial and temporal spread, that is, an epidemic would be thought to spread less and more slowly than it actually does.

Parameters in the model were estimated using data from a range of sources: those for transmission between farms were estimated from data for northern Europe in 2006, while those for transmission within farms were obtained from the published literature (Table 2; see also [17]). Wherever possible the estimates were derived for UK *Culicoides* spp. or for the BTV-8 epidemic in northern Europe, but some estimates were only available for other species or outbreak areas (see references in Table 2).

Data from the BTV outbreak in GB during autumn 2007 were not used to parameterise the model. Despite this, the model yields an adequate fit to the observed outbreak in GB, both in terms of the incidence of reported cases (Figure 3A) and the spatial extent of spread (Figure 4A). More refined estimates for certain parameters might be obtained by exploring epidemiological data available for GB, especially in relation to disentangling movement-based transmission from that due to vector dispersal in the spread of BTV between farms. However, it is worth noting that this is a relatively small data set (125 affected holdings). Of these 125 holdings, 42 were detected through clinical surveillance, but others were identified through serological surveillance (23) or pre-movement testing (60), which increases uncertainty on their likely date of infection.

A second area of uncertainty in the model relates to the biting rate of *Culicoides* vectors on ruminant hosts and how this changes over space and time. In our modelling approach, we set a maximum biting rate for each (affected) farm by sampling from a plausible range for this parameter and then allowed the biting rate to vary over time in response to changes in temperature (equation (1); Table 2) (cf. [32]). However, this approach does not allow for potentially systematic variation in the biting rate in response, for example, to climatic differences or husbandry practices.

Spatial and temporal variation in vector abundance (one component of the biting rate; cf. equation (1)) can be predicted by combining data from trap catches with satellite imagery [32]–[34]. However, there are problems interpreting trap catch data and their relationship with biting rates on host species. Although it is possible to relate trap catches to biting rates for certain *Culicoides* spp. (for example, carbon dioxide-baited suction traps for *C. sonorensis*, the principal North American vector of BTV [35]), there is evidence that this is not the case for European vector species and trapping methods [36], [37]. Finally, there are potential species-level differences amongst *Culicoides* vectors in terms of their distribution and competence (see [38] for a review), which could impact on the transmission dynamics of BTV, but which are not sufficiently well understood to be included in the model.

In the longer term, more complex and realistic models of transmission of BTV are needed, which include separate transmission routes and seasonal vector dynamics, as well as multiple vector species and virus strains. The epidemiological model presented here constitutes an ideal framework, which is highly flexible and can incorporate these aspects. However, basic but fundamental information to parameterise these additional components is missing and collecting these data should be made a priority.

## Supporting Information

Figure S1 Location of 19 meteorological stations in Great Britain. Hourly temperature records for 2007 were extracted for each station and used as inputs in a model for the transmission of bluetongue virus within and between farms in Great Britain. Each farm used temperature records from its nearest meteorological station.
(0.29 MB TIF)

Figure S2 Hourly temperature records for 2007 for 19 meteorological stations. Records are shown for each meteorological station shown in Figure S1 in order from the southernmost to the northernmost station.
(1.24 MB TIF)
