# Supplementary figures and images for: A Modeling Framework to Describe the Transmission of Bluetongue Virus within and between Farms in Great Britain
Source: PLoS One. 2009 Nov 5;4(11):e7741. doi: 10.1371/journal.pone.0007741 (PMC2767512; doi:10.1371/journal.pone.0007741)

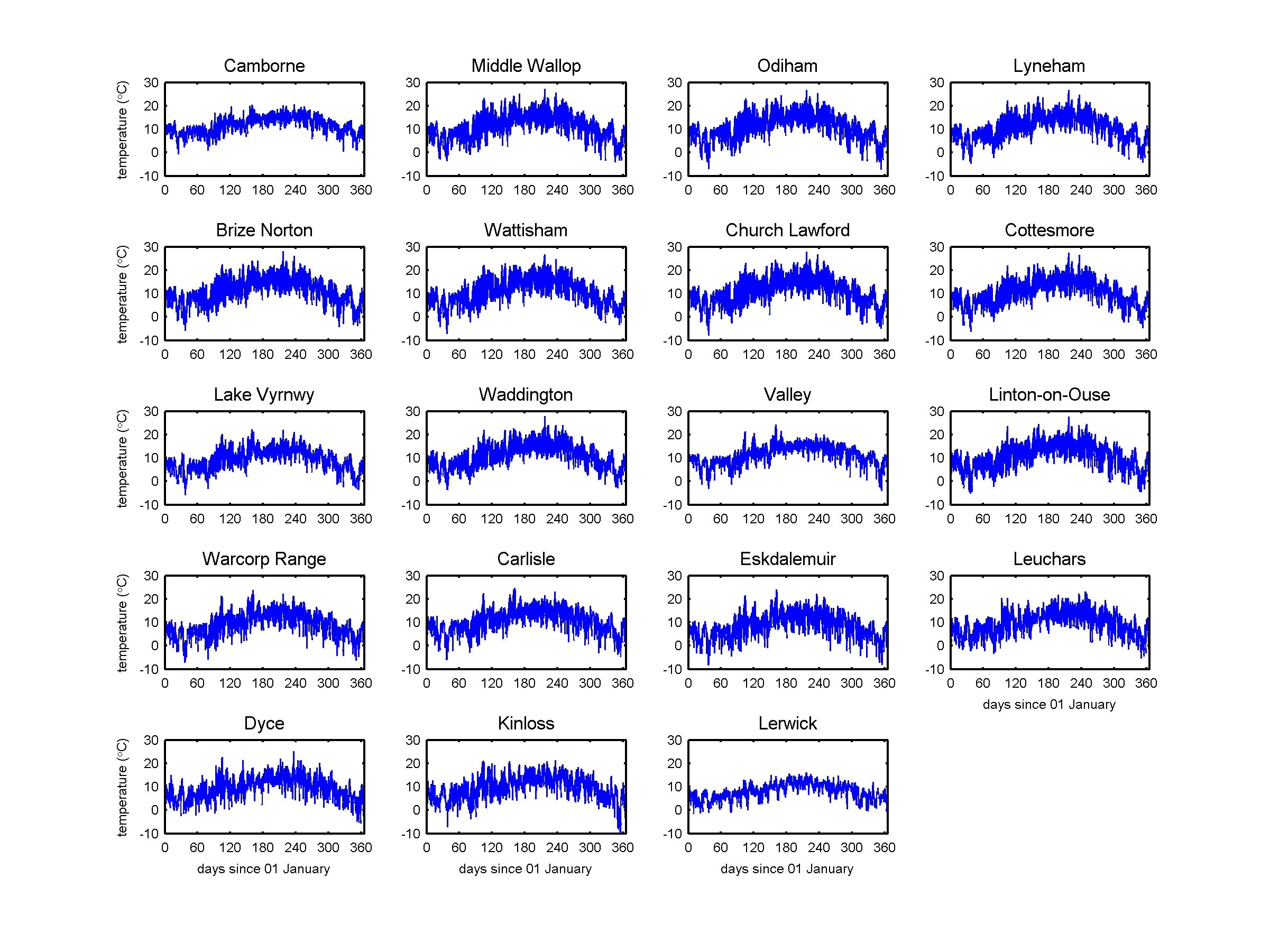

Supplement: Figure S2 — Hourly temperature records for 2007 for 19 meteorological stations. Records are shown for each meteorological station shown in Figure S1 in order from the southernmost to the northernmost station. (1.24 MB TIF) [file pone.0007741.s002.tif]
